# A Novel Function for the *Streptococcus pneumoniae* Aminopeptidase N: Inhibition of T Cell Effector Function through Regulation of TCR Signaling

**DOI:** 10.3389/fimmu.2017.01610

**Published:** 2017-11-27

**Authors:** Lance K. Blevins, Derek Parsonage, Melissa B. Oliver, Elizabeth Domzalski, W. Edward Swords, Martha A. Alexander-Miller

**Affiliations:** ^1^Department of Microbiology and Immunology, Wake Forest School of Medicine, Winston-Salem, NC, United States; ^2^Department of Biochemistry, Wake Forest School of Medicine, Winston-Salem, NC, United States

**Keywords:** pneumococcus, T cell, aminopeptidase, immune regulation, signaling

## Abstract

*Streptococcus pneumoniae* (*Spn*) causes a variety of disease states including fatal bacterial pneumonia. Our previous finding that introduction of *Spn* into an animal with ongoing influenza virus infection resulted in a CD8^+^ T cell population with reduced effector function gave rise to the possibility of direct regulation by pneumococcal components. Here, we show that treatment of effector T cells with lysate derived from *Spn* resulted in inhibition of IFNγ and tumor necrosis factor α production as well as of cytolytic granule release. *Spn* aminopeptidase N (PepN) was identified as the inhibitory bacterial component and surprisingly, this property was independent of the peptidase activity found in this family of proteins. Inhibitory activity was associated with reduced activation of ZAP-70, ERK1/2, c-Jun N-terminal kinase, and p38, demonstrating the ability of PepN to negatively regulate TCR signaling at multiple points in the cascade. These results reveal a novel immune regulatory function for a bacterial aminopeptidase.

## Introduction

The gram-positive bacterium *Streptococcus pneumoniae* (*Spn*) is the most common causes of fatal bacterial infections globally, with the Centers for Disease Control (1) estimating that ~900,000 Americans contract pneumococcal pneumonia each year. These infections result in greater than 400,000 hospitalizations with 5–7% of individuals succumbing to disease (2, 3). At the global level, the World Health Organization (4) estimates there are ~14 million cases of serious pneumococcal disease and more than 1.6 million people die each year from invasive pneumococcal disease (IPD) (4). Following introduction of the pneumococcal conjugate vaccine, the overall incidence of pneumococcal disease decreased (5). However, the prevalence of serotypes not covered by current conjugate vaccines is on the rise (6, 7). Our ability to effectively treat IPD is further complicated by the reported global rise of antibiotic resistant strains (8, 9). These challenges demonstrate that *Spn* remains a significant human health concern worldwide.

*Streptococcus pneumoniae* lives a dichotomous lifestyle within the host. The bacteria can persist in a typically asymptomatic carriage state (10), which is critical for transmission as well as the initiating event that leads to a more invasive phenotype in which the bacteria can ascend the Eustachian tube to take up residence in the inner ear (otitis media), or enter the blood stream (bacteremia), lungs (pneumonia), or meninges (meningitis) (10). While the nasopharynx of healthy adults is variably colonized with *Spn* (1), younger individuals exhibit frequent colonization with up to 60% of school-aged children testing positive (1).

Invasive pneumococcal disease signals the transition of the bacteria from a semiquiescent, asymptomatic state to one marked by rapid growth and dispersion as well as increased disease and mortality (11). The invasion of a relatively sterile tissue by *Spn* is marked by increased production of virulence factors such as capsule and pneumolysin (PLY) (11). Further, *Spn* increases autolysis and fratricide of neighboring pneumococci during this time (12, 13). This most likely facilitates invasion through increased inflammation of the host tissue, due in part to the release of PLY, which can directly stimulate the production of the proinflammatory cytokine tumor necrosis factor α (TNFα) through Toll-like receptor 4 signaling. Host inflammation is further increased by the release of cell wall that occurs during autolysis (11, 14).

The signals that dictate the transition from the carriage state to an invasive phenotype are poorly understood and heavily debated. However, clinical, epidemiological, and experimental data show that a preceding or concurrent viral infection is highly associated with this transition (15–18). A study by Heikkinen et al. demonstrated that the presence of a viral infection could be detected with highly sensitive PCR based assays in the nasopharyngeal secretions of 90% of children with acute otitis media (19). The presence of a respiratory viral pathogen has been associated with IPD in both children and adults (20, 21).

In humans and in mice, a significant portion of the antiviral immune response in respiratory mucosal spaces is mediated by CD8^+^ T cells. Effector and memory CD8^+^ T cells are readily found in these sites, e.g., lung airways (22, 23) and the nasopharyngeal space (24, 25). Both effector and memory CD8^+^ T cells rely on their ability to produce cytokines and/or kill cells to clear virally infected cells and to limit viral spread (26, 27). Considering that studies in multiple mouse strains have reported that both effector and memory T cells retain an activated phenotype in tissues (i.e., CD69^+^CD25^+^) (22, 25), it is important to consider that in the context of either pneumococcal carriage or IPD, there are significant opportunities for the colocalization of *Spn* and CD8^+^ T cells. As such, these cells are potential targets for immune regulation by pneumococcal components.

Our previous studies of *Spn* and influenza A virus (IAV) coinfection demonstrated that high levels of bacteria were associated with a significant decrease in the overall size of the cytokine-producing IAV-specific CD8^+^ T cell response (28). This decrease was the result of both increased death and unexpectedly, a lack of IFNγ and TNFα producing capabilities in the remaining effectors (28). The failure to produce IFNγ could not be restored by phorbol 12-myristate 13-acetate (PMA) and ionomycin (IONO) stimulation, which bypasses the membrane proximal steps of T cell receptor signaling (28). These findings strongly suggested that in areas where high bacterial burden is present concomitant with CD8^+^ T cells, there is significant potential for adverse effects on the CD8^+^ T cell response. Based on these findings, we hypothesized that *Spn* may produce unknown factors that have the ability to directly modulate CD8^+^ T lymphocytes through the negative regulation of effector function.

To directly test the possibility that *Spn* modulates CD8^+^ T cell effector function, we established a tractable system using peptide restimulation of antigen-specific CD8^+^ T cells in the presence of pneumococcal lysate. We observed active inhibition of cytokine production and release of cytolytic granules. No effects on T cell survival were found, demonstrating that functional impairment was not due to death induced by PLY. The inhibitory component in the lysate was identified as the pneumococcal enzyme aminopeptidase N (PepN). The ability of PepN to suppress cytokine production by CD8^+^ T cells correlated with reduced levels of phosphorylated Zeta-chain-associated protein kinase 70 (ZAP-70), extracellular-signal-regulated kinases 1 and 2 (ERK1/2), c-Jun N-terminal kinase (JNK), and the p38 mitogen-activated protein kinase. Importantly, the ability of PepN to inhibit T cell effector function was independent of its enzymatic activity, demonstrating a novel activity for this protein. Our study marks the first report of a bacterial aminopeptidase modulating T cell effector function and in addition elucidates a novel role for aminopeptidases in immune evasion by bacterial pathogens.

## Materials and Methods

### Bacterial Strains, Plasmids, Culture Conditions, and Reagents

Bacterial strains and plasmids used in this study are listed in Table 1. *Spn* was grown in Brain-Heart Infusion (Difco) broth supplemented with 10% heat-inactivated horse serum (Gibco) and 10% catalase (3 mg/ml) to an OD_600_ of 0.8, correlating to approximately 1 × 10^8^ CFU/mL. Broth cultures were mixed 1:1 with a 50% glycerol solution and frozen at −80°C for future use. *B. pertussis* strains were maintained on Bordet–Gengou agar (BG) supplemented with 10% defibrinated sheep blood. Liquid cultures were grown in Stainer–Scholte broth with heptakis (2,6-di-*O*-methyl-β-cyclodextrin) (29). *Escherichia coli* strains were grown in Luria–Bertani medium. As necessary, the growth media were supplemented with appropriate antibiotics, chloramphenicol (Cm, 10 or 50 µg ml^−1^), kanamycin (Km, 25 µg ml^−1^), and streptomycin (Sm, 100 µg ml^−1^).

**Table 1:** Bacteria used in study.

Bacteria	Description; serotype	Reference or source
***Streptococcus pneumoniae***		
D39	Lab strain; 2	Avery et al. (30)
TIGR4	Lab strain; 4	Lanie et al. (31)
EF3030	Lab strain; 19F	Briles et al. (32)
MNZ1113	Clinical isolate; Null	Hiller et al. (33)
BG12740	Clinical isolate; 6A	D. Briles, UAB
EF6796	Clinical isolate; 6A	D. Briles, UAB
L8-2044	Clinical isolate; 15C	D. Briles, UAB
13678	Clinical isolate; 6B	R. Dagan, BGU Israel
10955	Clinical isolate; 15	R. Dagan, BGU Israel
16654	Clinical isolate; 23F	R. Dagan, BGU Israel
26968	Clinical isolate; 33	R. Dagan, BGU Israel
***Bordetella pertussis***		
BP536	Lab strain	Relman et al. (34)

### Preparation of Bacterial Lysates

Broth cultures were inoculated with *Spn, Bps*, or *E*. *coli* under conditions described above and cultured overnight. 1% w/v choline chloride (Sigma) was added to *Spn* cultures to limit bacterial autolysis. Following the culture period, bacteria were pelleted by centrifugation and washed 3–5× with ice cold PBS. Bacteria were disrupted by passaging 5× through a C3 Avestin emulsifier. Crude lysates were centrifuged (12,000 *g*) to pellet insoluble material. Protein was quantified with a BCA protein kit (Pierce). Lysates were aliquoted and stored at −80°C for future use.

### Influenza A/PR/8/34 (H1N1)

Virus stocks were grown and tittered [median egg infectious dose (EID_50_)] in 10-day-old fertilized chicken eggs (obtained from a local farm) eggs as described previously (35). Stocks were diluted in PBS, flash frozen, and stored at −80°C.

### Mice

The 8–10–week-old female BALB/c mice were purchased from The Jackson Laboratories. Mice were housed in a biosafety level 2 facility with *ad libitum* access to food and water.

### *In Vivo* Elicitation of NP-Specific Cytotoxic T Lymphocyte (CTL) and Establishment of Line

Mice were anesthetized with Avertin (2,2,2-tribromoethanol) by intraperitoneal (i.p.) injection. Virus (10^3^ EID_50_) was administered *via* the intranasal (i.n.) route in 50 µl of PBS. Mediastinal lymph nodes (MLNs) were harvested 8 days following IAV infection. Cells were isolated and co-cultured in enriched medium in the presence of irradiated splenocytes previously pulsed with 10^−7^ M NP_147–155_ peptide at a ratio of 1:10. 10% T-stim (Corning) was added as a source of IL-2. Cultures were restimulated weekly.

### Stimulation of T Cells and Analysis of Effector Function and Death

On day 6 or 7 postweekly stimulation, cells were cocultured *in vitro* with 10^−6^ M NP_147–155_ peptide or PMA + IONO in the presence or absence of the indicated amount of lysate or PepN. Monensin and brefeldin A (BD Biosciences) were added to inhibit secretion of cytokines. For cells stimulated with peptide, BV420 conjugated NP_147–155_/K^d^ tetramer (graciously supplied by the NIH tetramer facility) was included during the stimulation. This allowed tetramer labeling that otherwise may have been hampered as a result of TCR downregulation that occurs with stimulation. Anti-CD107a antibody (Biolegend) was also included during the stimulation period to capture granule release. Following the 5 h stimulation period, samples were stained with antimouse CD8α (Biolegend) and BV420 conjugated NP_147–155_/K^d^ tetramer (necessary to identify antigen specific cells in the non-stimulated samples). Cells were then fixed and permeabilized (Cytofix/Cytoperm kit, BD Biosciences) followed by incubation with antibodies specific for IFNγ (Biolegend) and in some cases TNFα (Biolegend). Where indicated, data were normalized to % inhibition by the following: [%IFNγ^+^/%IFNγ^+^(No Lysate/PepN)] × 100 = %Functional; %Inhibition = 100 − %Functional. To determine cell viability, cells were incubated with 7-AAD (Biolegend) following antibody staining. Cells were then washed extensively prior to fixation and permeabilization.

For analysis of TCR signaling, cells were stimulated with peptide or PMA + IONO as indicated for 15 min. For the induction of STAT1 phosphorylation, CTL were stimulated for 15 min with 5,000 Units of Universal Type 1 IFN (PBL). Following the stimulation period cells were fixed in 2% paraformaldehyde for 10 min at 37°C. Cells were then washed, stained with anti-CD8 antibody, and fixed/permeabilized with True-Nuclear Transcription Factor Staining kit (Biolegend) per the manufacturer’s instructions. The following antibodies were used: anti-ERK1/2 (Santa Cruz), antiphosphorylated ERK1/2 (ebioscience), antiphosphorylated p38 (ebioscience), antiphosphorylated ZAP-70 (BD Biosciences), antiphosphorylated JNK (BD Biosciences), antiphosphorylated STAT1 (BD Biosciences), and antiphosphorylated mammalian target of rapamycin (mTOR) (ebioscience).

### Fractionation of *Spn* Lysates

Anion Exchange chromatography: *Spn* lysate was filtered and NaCl (Sigma) added to a final concentration of 10 mM. Lysate was then loaded onto a Q Sepharose HP column (GE Lifesciences). Proteins were eluted with an increasing gradient of NaCl (10 mM to 1 M) and fractions collected. Hydroxyapatite chromatography: Fractions from the previous step that contained inhibitory activity in our functional assay were pooled. Potassium phosphate (KPi, Sigma) was added to pooled fractions to a final concentration of 10 mM. These fractions were loaded onto a hydroxyapatite column (Macro-Prep ceramic hydroxyapatite resin, type 1, 40 µM, Bio-Rad). Proteins were eluted with a 10–500 mM KPi gradient; fractions were collected and tested for inhibition of T cell effector function.

### PCR, DNA Sequencing, and Cloning of Bacterial PepN

To clone the *pepN* gene, the sequence of *Spn* strain EF3030 *pepN* was determined as follows: genomic DNA was subjected to PCR amplification using primers designed based on intergenic identity of strains D39, AP200, R6, and TIGR4 (Table 2). PCR products were purified with a Wizard PCR clean-up kit (Promega) and sequencing performed by Eton Bioscience Inc. (San Diego, CA, USA). DNA sequences were analyzed with ApE software (36) and the Basic Local Alignment Search Tool (http://www.ncbi.nlm.nih.gov/BLAST/).

**Table 2:** Primers used for sequencing *pepn*.

Primer name	Sequence
pepNF1	cagaagtttatcaaaccaacccc
pepNF2	gaccatccagatcatcttaaac
pepNF3	cctgaaagcaaggttgttttag
pepNF4	gacaacatgacagggatttac
pepNF5	cgcccttcctgacttctc
pepNF6	ggtttgcacgcctactttg
pepNF7	gcagacttgcttccagttc
pepNF8	ctgattacttcttggaaggac
pepNF9	cggaaatgcataaataagcc
pepNR1	gggaaccacttttgcagag

Following confirmation of *pepN* gene sequence (submitted: GenBank KX522575), the gene was amplified from genomic DNA using primers to introduce a 5′ *BamHI* and a 3′ *XhoI* cut site [pepNF1-BamHI (cccggggatccatgcaagcagttgaacat), pepNR1-XhoI (cccgggctcgagttatgcatttccgtattg)]. PCR products were digested with *BamHI* and *XhoI* restriction enzymes (NEB) and purified. The *pepN* gene insert was then ligated into the pTHC-Δ*P* expression vector that enables cloning in frame with a His tag [for reference see Ref. (37)] using T4 DNA ligase (NEB). The resulting mixture was then transformed into XL-1 Blue *E*. *coli* (Stratagene), plated under selective conditions and colonies screened for pepN containing plasmid by double restriction digest with *BamHI* and *XhoI*.

### PepN Overexpression and Purification

*Escherichia coli* carrying *Spn pepN* were grown overnight under conditions to induce overexpression of PepN. The following day bacterial lysate was prepared as described above with the inclusion of 1 mM protease inhibitors (AEBSF, aprotinin, bestatin, E-64, leupeptin, and pepstatin A) (Pierce). Aliquots of lysate were run on a denaturing Bis-Tris SDS-PAGE resolving gel consisting of 10% acrylamide (BioRad). Proteins were visualized by staining with GelCode Blue Safe Protein Stain (Thermo). His-tagged PepN was purified by passage of lysate over 10 mL of Nickel Sepharose (GE Life Sciences). Bound protein was washed 3× with Tris buffer containing 50 mM sodium phosphate (Sigma), 10% glycerol (Fischer), 10 mM imidazole (Sigma), and 0.25% Tween-20 (Sigma). Tagged protein was eluted with buffer containing 150 mM imidazole. Following purification, PepN was dialyzed against PBS overnight at 4°C with PreScission protease (GE Life Sciences) to cleave the His-tag and remove any residual buffer components. The following day, PepN concentration was determined by BCA (Pierce) and then aliquoted and stored at −80°C for later use.

### Quantitation of Cellular mRNA Levels by Real-time PCR

Following a 5-h peptide stimulation in the presence of PepN, lymphocyte RNA was isolated using an RNeasy RNA isolation kit (Qiagen). cDNA was synthesized from mRNA by reverse transcription using Superscript III RT kit (Invitrogen) and random primers (Promega). For IFNγ and GAPDH mRNA analysis, commercially available Taqman primer-probe sets specific for the gene targets were used. RT-PCR (qRT-PCR) was performed using the Applied Biosystems 7500 real-time PCR system. Raw data values were normalized to GAPDH mRNA levels.

### Mutagenesis of PepN

The zinc-binding domain of PepN was rendered incapable of binding zinc by introducing the following mutations: His292Ala, Glu293Ala, and His296Ala. Site directed mutagenesis was performed by Genscript as a fee for service.

### Quantification of Peptidase Activity

To quantify the peptidase activity of PepN and PepN^Met−^, 1 µg of each enzyme was incubated with 200 µM of lys-AMC (BACHEM) at 37°C for 2 h. Following incubation, samples were excited in the 355 nm range and signal was detected in the 460 nm range using a 96-well PolarStar plate reader (Omega, BMG LABTECH). 1 µg of trypsin (Gibco) was used as a positive control.

### Statistical Analysis

Data analysis was performed using GraphPad Prism (GraphPad Software). Significance was determined using either a two-tailed Student’s *t*-test or a repeated measure ANOVA with a Tukey’s posttest as appropriate.

## Results

### Stimulation of NP-Specific CD8^+^ CTL Line in the Presence of Pneumococcal Lysate Inhibits Effector Function

**Figure 1:**
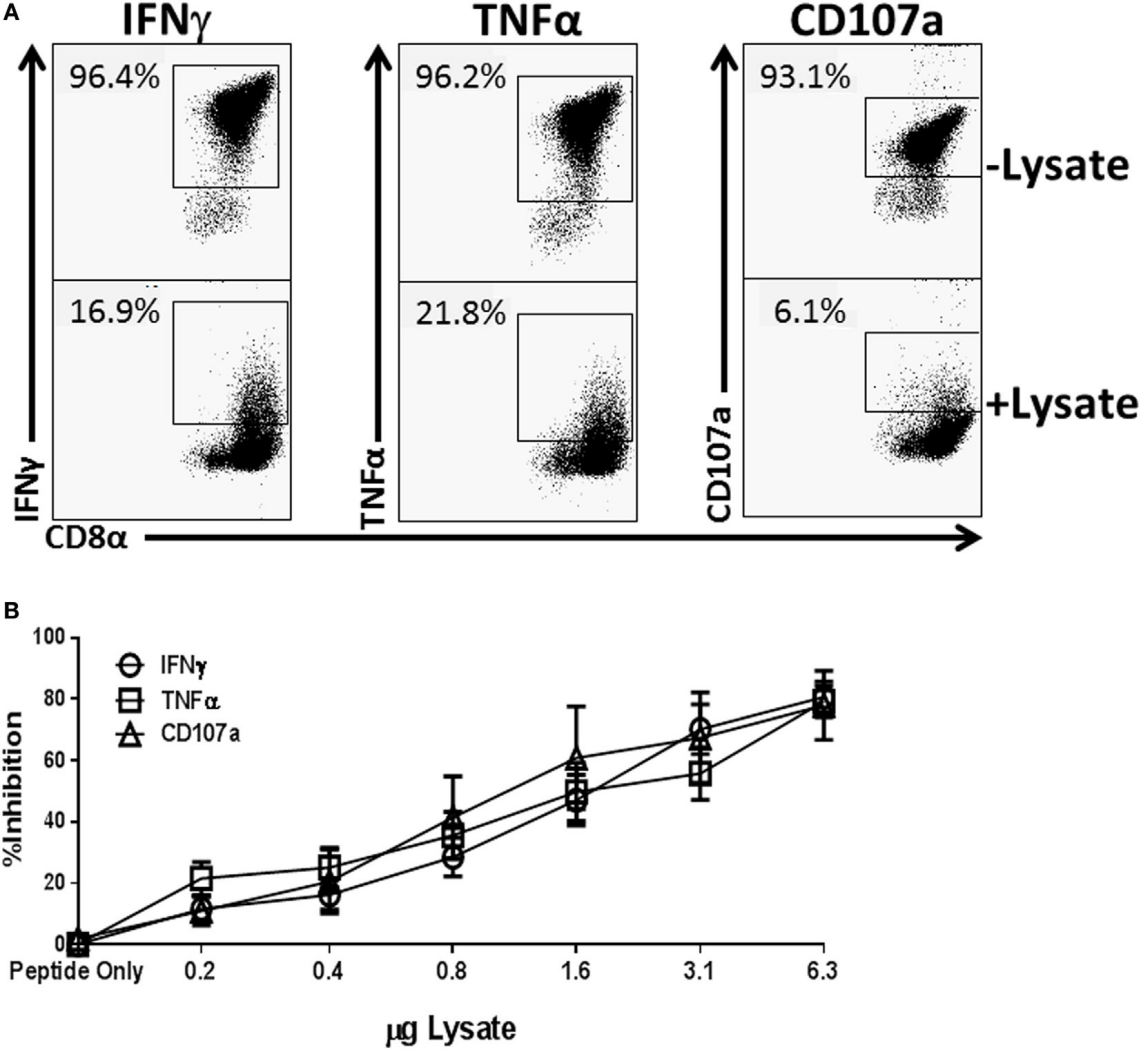
Treatment of NP-specific CD8^+^ cytotoxic T lymphocyte (CTL) with EF3030-derived lysate inhibits the production of IFNγ and tumor necrosis factor α (TNFα) and the release of cytolytic granules. NP_147–155_-specific CTL was stimulated with peptide in the presence of increasing amounts of *Spn* lysate. Anti-CD107a antibody was included in the stimulation phase to identify cells releasing lytic granules. Following stimulation, cells were stained for CD8, IFNγ, and TNFα. Data shown are pregated on live CD8^+^ CTL. The percent inhibition refers to loss of the effector function noted in the legend. **(A)** Representative flow plots. **(B)** Averaged data ± SEM from eight independent experiments assessed using two independently generated NP-specific CTL lines.

To assess the potential for *Spn* to directly modulate an effector T cell response, we employed an *in vitro* model that allowed for rapid and high throughput assessment on CTL function. We produced a lysate from the 19F serotype strain (EF3030) used in our previous *in vivo* studies (28) by overnight growth and subsequent mechanical disruption. To test the ability to regulate T cell function, increasing amounts of lysate were added at the time of NP-peptide restimulation of a CD8^+^ NP-specific CTL line. In the absence of lysate over 95% of the cells produced IFNγ and TNFα and were cytolytic as evidenced by CD107a positivity (Figure 1A). However, as the amount of *Spn* lysate present during peptide restimulation increased, we observed a dose-dependent inhibition of IFNγ and TNFα production as well as cytolytic granule release (Figure 1A representative flow plots at 3 µg, Figure 1B averaged data across the dose response). These data demonstrate the potential for bacterial components from *Spn* to directly inhibit CD8^+^ T cell effector function in a dose-dependent manner.

### *Spn* Lysate Inhibits Effector Function of Influenza Specific Effector CD8^+^ T Cells Generated *In Vivo*

**Figure 2:**
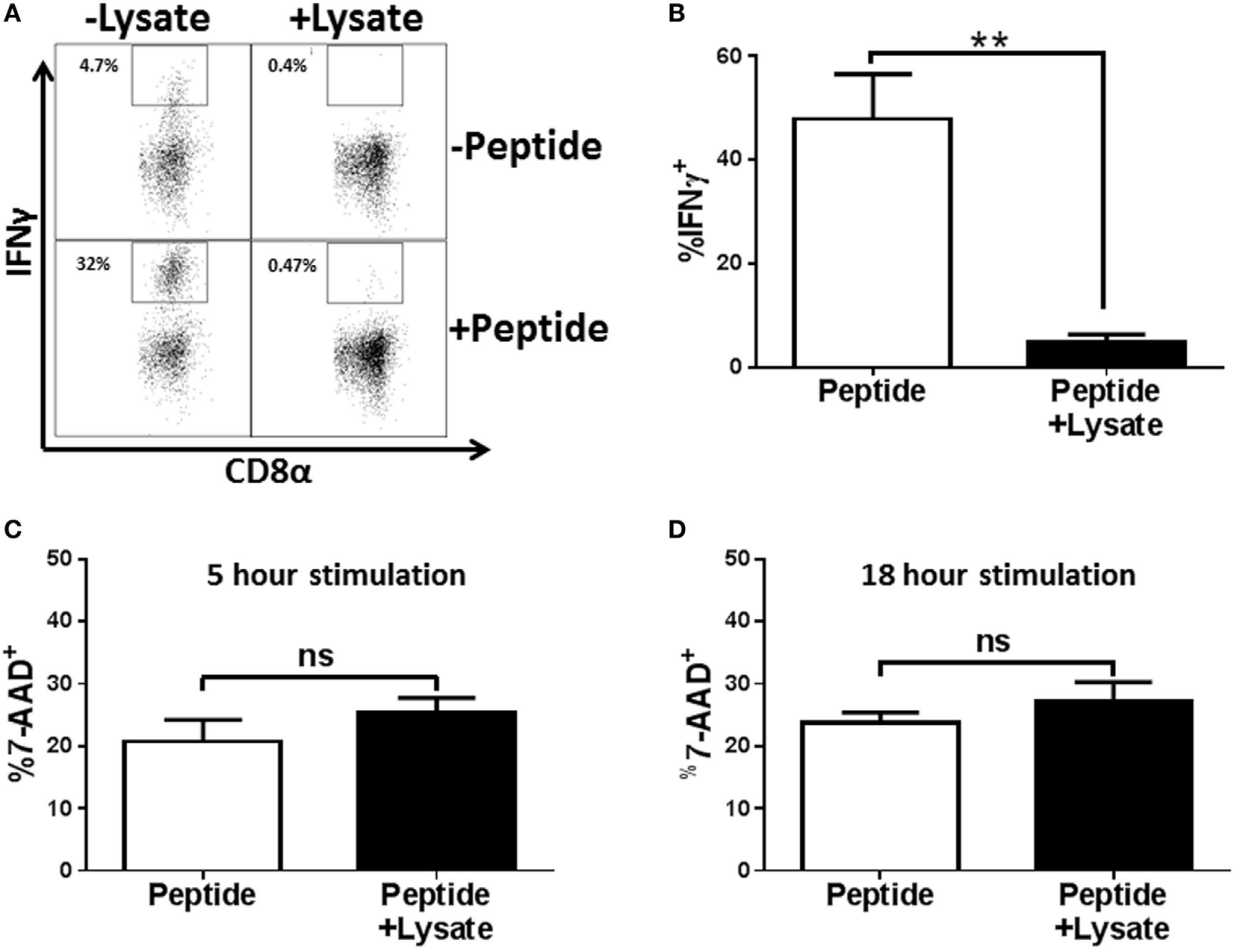
EF3030-derived lysate inhibits *in vivo* generated NP-specific CD8^+^ T cell effectors in the absence of cytotoxicity. Mice infected with influenza virus were euthanized on day 8 post infection. Mediastinal lymph nodes (MLNs) were harvested and cells stimulated *ex vivo* with influenza NP_147–155_ peptide and the indicated amount of lysate for 5 h **(A–C)** or 18 h **(D)**. Following stimulation, cells were stained for CD8, tetramer, IFNγ, and 7-AAD. Data shown are pre-gated on CD8^+^ tetramer^+^ cells. **(A)** Representative flow plots. **(B)** Averaged data from 5 independent experiments evaluating IFNγ production. Cell viability was assessed after 5 h **(C)** or 18 h **(D)** of peptide stimulation. Averaged data ± SEM from four or five independent experiments are shown in **(C,D)**, respectively. **p < 0.01.

To extend our results to effectors that had not been subjected to multiple cycles of *in vitro* stimulation, BALB/c mice were infected with the H1N1 influenza virus A/Puerto Rico/8/1934 and cells from the lung draining MLNs were restimulated *ex vivo* with peptide in the presence of NP_147–155_/K^d^ tetramer (this approach negated any issues with adequate tetramer labeling that could result from peptide-mediated TCR internalization). Following restimulation, cells were stained with anti-CD8, anti-IFNγ, and tetramer (to identify antigen-specific cells in the non-stimulated sample). As shown in Figure 2A, we detected a robust IFNγ response with peptide restimulation that was severely diminished in the presence of lysate. On average approximately 47.9% of CD8^+^Tet^+^ cells from the MLN of infected mice produced IFNγ compared to 4.7% of cells treated with *Spn* lysate (Figure 2B), representing a 10.2-fold reduction in IFNγ-producing cells. One explanation for this effect was that IFNγ production was impaired in cells treated with lysate as a result of killing by PLY, the cytotoxin present in *Spn* (38, 39). To assess death mediated by the lysate, effectors from the MLN of influenza infected mice were stimulated in the presence of lysate and cell viability determined by exclusion of 7-AAD. As shown in Figure 2C, cells stimulated with 3 µg of *Spn* lysate, a dose that has strong inhibitory function for cytokine production, had similar levels of 7-AAD positivity as cells stimulated in the absence of lysate. These data establish that a dose that was maximally inhibitory is not toxic to the CD8^+^ effector cells. To ensure that cells treated with lysate were not impaired because they were in the early stages of death, cells were stimulated for 18 h in the presence or absence of *Spn* lysate. Similar to what we observed with the 5-h exposure, there was no difference in death in treated versus non-treated cells (Figure 2D). These data rule out the induction of death as a mechanism to explain the lack of effector function in cells treated with lysate.

### Inhibition of CTL Function Is a Property Specific to Pneumococcal Lysates

**Figure 3:**
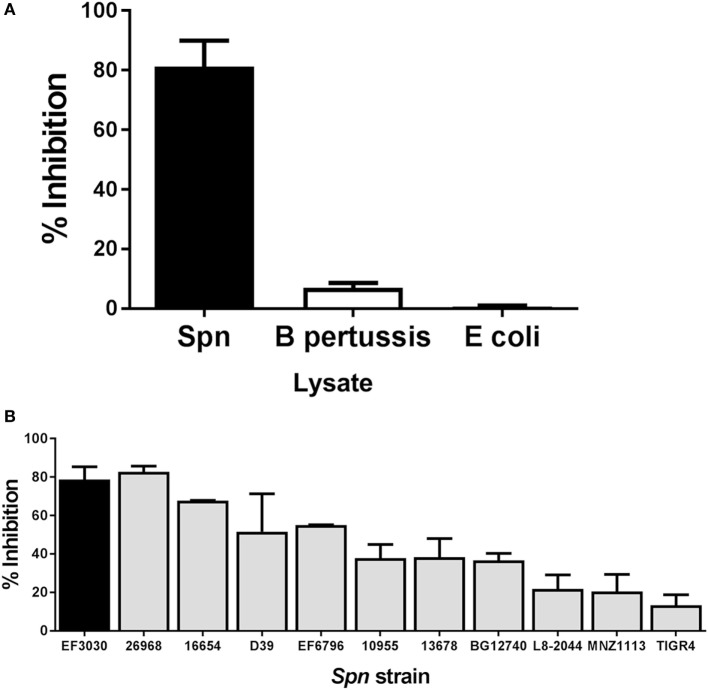
Inhibition of cytotoxic T lymphocyte (CTL) function is a property of pneumococcal, but not bacterial lysates in general. NP-specific CTL were stimulated *in vitro* in the presence of lysates derived from other bacterial species or a panel of *Spn* strains. Averaged data ± SEM from three to six independent experiments **(A)** or two to eight independent experiments **(B)** are shown. Percent inhibition reflects loss of IFNγ production. Experiments were conducted using a minimum of two independently derived CTL lines.

Bacterial pathogens produce a broad array of bacterial toxins (40). To determine whether the inhibitory effect observed following addition of *Spn* lysate could be attributed to a non-specific effect of adding bacterial lysate to T cells during stimulation, we generated lysates from the respiratory pathogen *Bordetella pertussis* (BP536) and a laboratory strain of *E. coli* (XL-1 Blue). Lysates from overnight cultures of these bacteria were prepared in the same manner as the *Spn* lysate. NP-specific CTL were then restimulated with peptide in the presence or absence of these bacterial lysates. While T cells stimulated with peptide in the presence of *Spn* lysate showed 80% inhibition in cytokine production (Figure 3A) stimulation with the *B. pertussis* or *E. coli* lysate showed minimal inhibition (Figure 3A). These data support the hypothesis that the T cell inhibitory activity was not a general property of bacterial lysates, but rather was mediated by a pneumococcal product(s).

### Lysates Derived from a Panel of Pneumococcal Strains Exhibit a Range of Inhibitory Potential

We next tested the hypothesis that the ability to inhibit T cell effector function was a general property of *Spn*, i.e., that lysates generated from strains encompassing a variety of serotypes exhibited this capability. *Spn* currently has over 90 known serotypes, which induce a disease encompassing a range of severity (41). We included in our panel pathogenic strains such as TIGR4 or D39 (capsular serotypes 4 and 2, respectively) as well as an unencapsulated strain, MNZ1113 (Table 1). Lysates from all strains were generated in the same manner as EF3030 lysates. Influenza-specific CTL were restimulated with peptide in the presence or absence of each lysate. As shown in Figure 3B, we found that all of the lysates were capable of inhibiting IFNγ production. Interestingly, there was divergence with regard to the degree of inhibition conferred by lysates from the different strains. The pattern did not correlate with any particular capsular serotype. These data establish the ability of multiple strains of *Spn* to inhibit CD8^+^ T cell responses.

### The Inhibition of Antigen Specific CTL Effector Function Is due to the Pneumococcal Aminopeptidase, PepN

**Figure 4:**
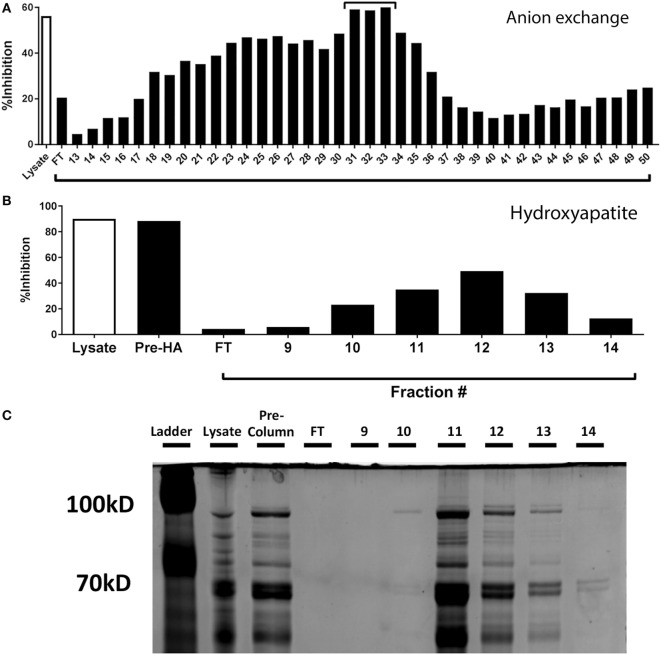
Purification and identification of the inhibitory protein from *Streptococcus pneumoniae* (*Spn*) lysates. **(A)** Proteins from EF3030-derived lysate were purified by anion exchange chromatography and protein containing fractions tested for their ability to inhibit IFNγ production. Total lysate served as a control. The fractions with the maximal inhibitory capability [denoted with a bar in **(A)**] were pooled and passed over a hydroxyapatite column. The ability of the collected fractions to inhibit IFNγ production by NP-specific CTL is shown in **(B)**. In addition to total lysate, the pooled fractions from the anion exchange column are shown (Pre-HA). Samples from B were run on a denaturing polyacrylamide gel and stained with Coomassie Blue protein stain **(C)**. The protein band with an intensity that correlated with the inhibitory activity is identified by an arrow.

To identify the component responsible for the inhibitory activity, we subjected the lysate to heat (100°C) and proteinase K treatment and found both of these treatments completely ablated the ability of the *Spn* lysate to inhibit IFNγ production by CTL (data not shown). Based on these results supporting the proteinaceous nature of the component, we passed the *Spn* lysate over an anion exchange column under constant flow. Fractions were collected every minute. Based on the elution pattern of protein, fractions 13–50 were tested for inhibitory activity in our peptide restimulation assay. Maximal inhibitory activity was observed in fractions 31–33 (Figure 4A). These fractions were pooled and further purified by passage over a hydroxyapatite column. The protein-containing fractions (9–14) were tested for inhibitory activity. The majority of activity was found in fractions 11–13 with maximal activity in fraction 12 (Figure 4B).

Fractions 11–13 were run on a denaturing polyacrylamide gel and proteins visualized by Coomassie staining. Each fraction contained multiple proteins, with the majority of protein eluting in fraction 11, despite maximal inhibition occurring with fraction 12 (Figure 4C). Upon close inspection, the intensity of a band at approximately 100 kDa (Figure 4C, arrow) was observed to be most correlative with inhibitory activity. This band was excised, extracted, and subjected to liquid chromatography and mass spectrometry to identify protein candidates, one of which was the pneumococcal metalloenzyme PepN.

To determine whether *Spn* PepN was in fact responsible for the inhibition of CTL effector function, the protein was produced in *E. coli*. PepN has been described in other closely related bacterial species, e.g., *Streptococcus thermophilus*, but the pneumococcal PepN has not been characterized to date. We designed primers in the intergenic regions surrounding the PepN gene based on genetic alignment of strains of pneumococcus for which sequences are available. Following PCR amplification and sequencing to confirm identity, the *pepn* gene was cloned from EF3030 into the *E. coli*-based protein overexpression vector pTHC-Δ*P*. This vector is engineered to add a sequence encoding a 6× His tag to the N terminus of the cloned gene. Following transformation, *E. coli* positive for the *pepn* containing vector were selected and cultured under conditions that induced the production of PepN.

**Figure 5:**
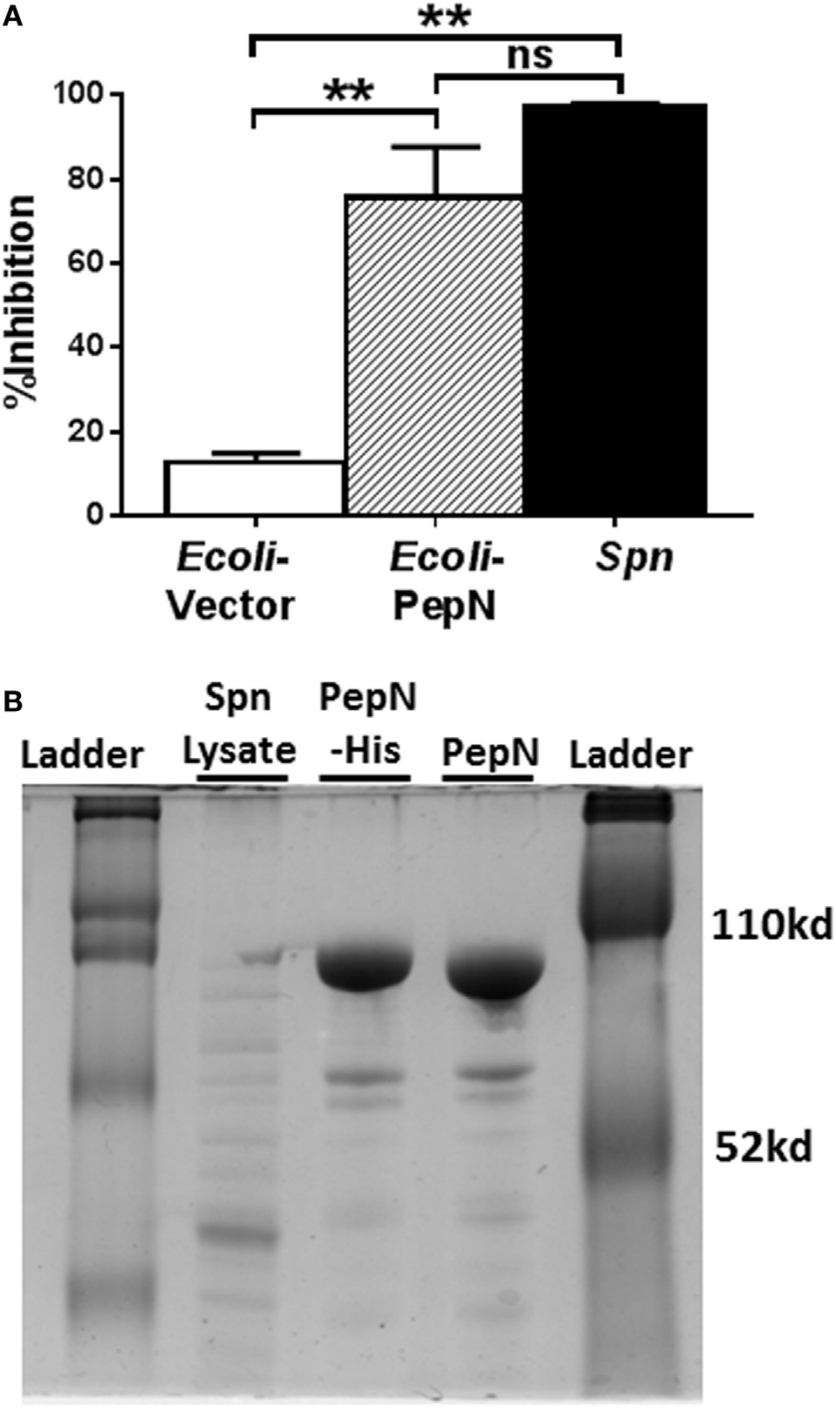
Lysate derived from aminopeptidase N (PepN)-His expressing *Escherichia coli* inhibits effector function. NP-specific cytotoxic T lymphocyte (CTL) was stimulated with peptide in the presence of 3 µg of lysate derived from *E. coli* transformed with either empty vector or the vector expressing PepN or with *Streptococcus pneumoniae* (*Spn*) lysate. **(A)** Percent inhibition of IFNγ production by *Escherichia coli* or *Spn* lysates (mean ± SEM). Data are from at least three independent experiments. A repeated measure ANOVA with a Tukey’s posttest was used to determine significance. **p < 0.01. **(B)** Polyacrylamide gel depicting S*pn* lysate, column purified PepN-His and PepN with His tag removed.

As a first step, we determined whether the lysate prepared from *E*. coli expressing PepN exhibited inhibitory activity. NP-specific CTL were stimulated with peptide in the presence of lysates from *Spn*, empty vector transformed *E. coli*, or *E. coli* expressing PepN-His. As expected, the lysate derived from *E. coli* transformed with the empty vector induced minimal inhibition of IFNγ (Figure 5A). In agreement with our previous results, significant inhibition of IFNγ production was observed following treatment with *Spn* lysate (Figure 5A, black bar). Addition of lysate prepared from *E. coli* expressing PepN also resulted in robust inhibition of cytokine production (Figure 5A, hatched bar), supporting the hypothesis that PepN mediates inhibition of CD8^+^ CTL effector function.

Given the evidence of PepN inhibitory activity in the lysate of *E. coli* transformed with the PepN vector, PepN was purified by passage over a nickel column. The His tag was subsequently cleaved. The purified proteins were assessed by gel electrophoresis (Figure 5B). The difference in mobility between the His-tagged protein (PepN-His) and the cleaved protein (PepN) indicated successful removal of the tag.

### Purified *Spn* PepN Inhibits NP-Specific CTL Effector Function

**Figure 6:**
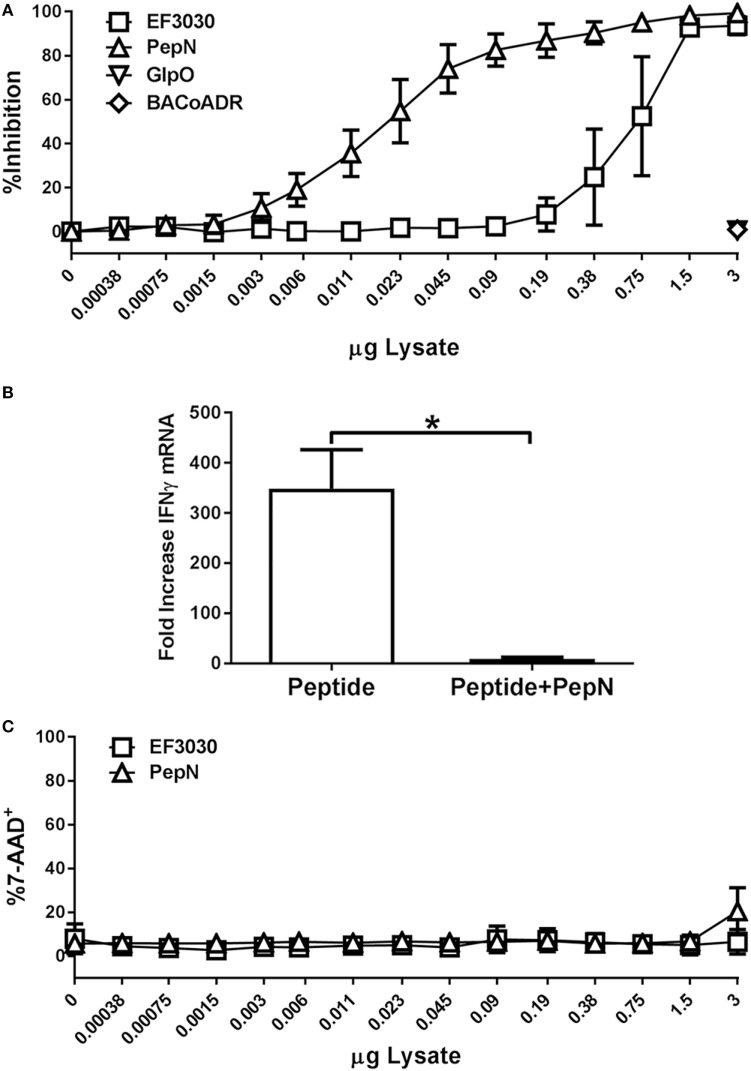
Purified aminopeptidase N (PepN) shows enhanced T cell inhibitory activity relative to *Streptococcus pneumoniae* (*Spn*) lysate and this effect is independent of toxicity. NP-specific cytotoxic T lymphocyte (CTL) were stimulated with peptide in the presence of increasing amounts of EF3030-derived lysate or purified PepN. Following stimulation, cells were stained with anti-CD8 and anti-IFNγ antibodies. 7-AAD was used to assess cell viability. **(A)** Mean ± SEM for PepN inhibition of IFNγ production. **(B)** Mean ± SEM of IFNγ mRNA following treatment of CTL with PepN. **(C)** Cell viability data are represented as the mean ± SEM. Data are from three independent experiments using at least two independently generated NP-specific CTL lines.

In order to establish that PepN from *Spn* was sufficient for mediating the inhibition of CTL effector function, we added titrated amounts of purified PepN versus *Spn* lysate during stimulation. To control for non-specific effects of adding purified *E. coli-*expressed protein, we used *Bacillus anthracis* coenzyme A-disulfide reductase (BACoADR) and l-α-glycerophosphate oxidase (GlpO), which were purified in a similar manner to PepN. As shown in Figure 6A, increasing amounts of *Spn* lysate resulted in a corresponding increase in inhibition of IFNγ production following peptide stimulation. The *Spn* lysate was capable of inhibiting approximately 50% of the response with 0.75 µg. In contrast, the purified PepN exhibited 50% inhibition of IFNγ production at 0.025 µg of PepN, which corresponds to an approximately 33-fold increase in the activity compared to the lysate. Inhibition was not a non-specific effect as the addition of 3 µg of BACoADR or GlpO did not alter the production of IFNγ (Figure 6A). The loss of IFNγ production was associated with a failure to produce IFNγ mRNA (Figure 6B). While peptide stimulation resulted in a 349-fold increase, message levels increased by only 9.6-fold when PepN was present, supporting regulation at the level of transcription.

To determine whether PepN treatment had cytotoxic effects in our T cell function assay, cells were stained with 7-AAD as previously described. As shown in Figure 6C, PepN did not induce a significant increase in 7-AAD positivity at any of the concentrations tested. These data show PepN-mediated inhibition occurs in the absence of cell death.

### PepN-Mediated Inhibition of IFNγ Production Is Independent of the Peptidase Activity of PepN

**Figure 7:**
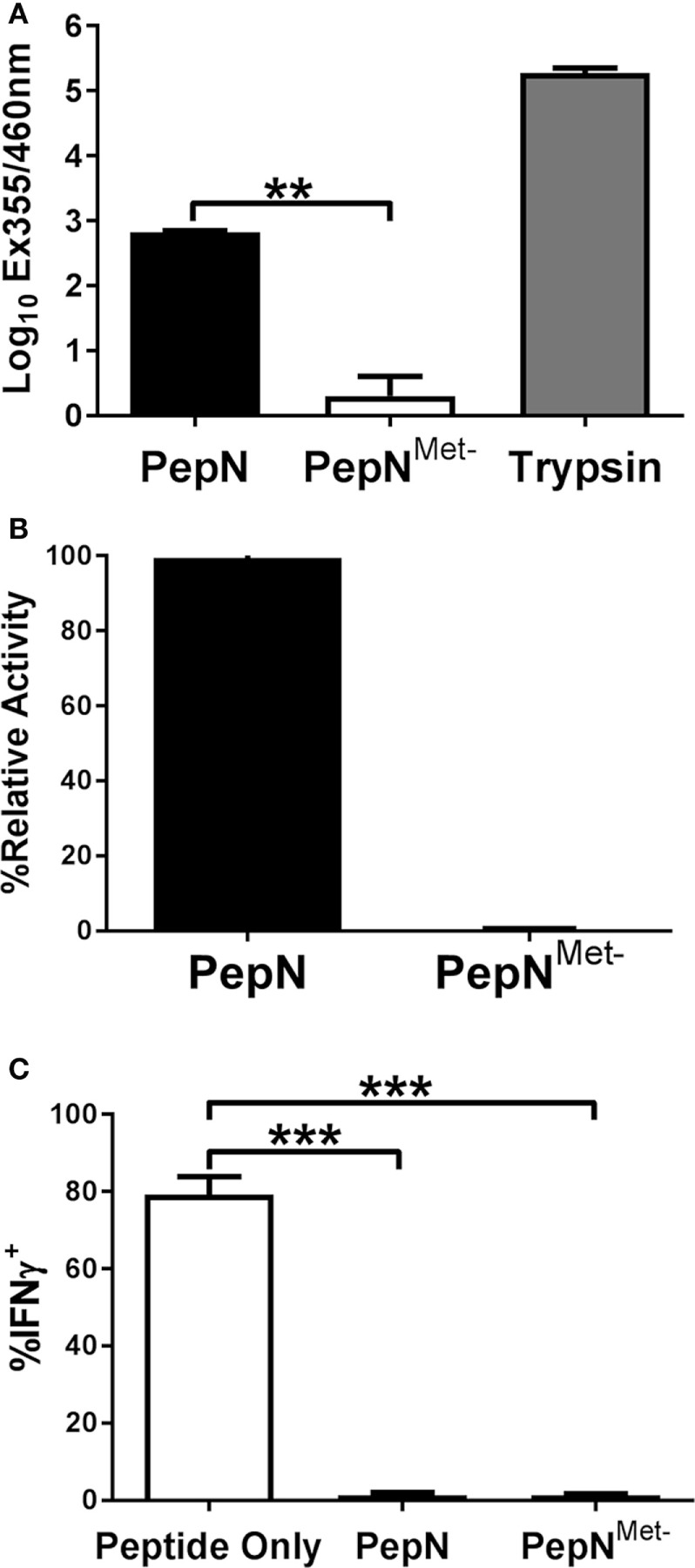
The ability of *Streptococcus pneumoniae* (*Spn*) aminopeptidase N (PepN) to inhibit NP-specific cytotoxic T lymphocyte (CTL) effector function is independent of its enzymatic activity. The enzymatic activity of wild-type PepN and PepN^Met−^ was tested by incubation with lys-AMC. Raw absorbance values from three independent experiments are shown in **(A)**. The average of values normalized to the level of activity observed using wild-type PepN (plotted as % relative activity) is shown in **(B)**. In **(C)**, NP-specific CTL were stimulated in the presence of PepN or PepN^Met−^. Cells were stained with anti-CD8 and anti-IFNγ antibodies following the stimulation period. Averaged data ± SEM from three independent experiments assessing the inhibitory activity of each protein is shown. **p < 0.01 and ***p < 0.001.

We hypothesized the ability of PepN to inhibit IFNγ was dependent on its known enzymatic activity. Like other metalloproteases, PepN activity relies on metal ion interaction with the binding pocket in the enzymatic domain of the protein (42). In order to test the dependence on peptidase activity, we generated a mutant PepN that was incapable of binding zinc as a result of mutation of His292Ala, Glu293Ala, and His296Ala (PepN^Met−^) (43). Following generation of the PepN mutant, we tested enzymatic activity by measuring the cleavage of lysine from an aminomethylcoumarin (AMC) substrate. Trypsin served as a positive control. As shown in Figure 7A, trypsin treatment resulted in robust hydrolysis of lysine-AMC. Purified PepN also hydrolyzed the lysine-AMC substrate. In contrast, PepN^Met−^ failed to hydrolyze the substrate (Figure 7A). When normalized to the relative activity of wild-type PepN, the metal negative mutant exhibited only 0.3% activity (Figure 7B) confirming that the mutations in the zinc-binding domain rendered PepN enzymatically inactive.

To test the inhibitory activity of the PepN mutant, CTL were stimulated in the presence or absence of PepN^Met−^ and IFNγ production was measured. As expected, PepN inhibited nearly all IFNγ production induced by peptide stimulation (Figure 7C). Surprisingly the enzymatically inactive PepN exhibited similar inhibitory activity. Thus, the ability of PepN to inhibit CTL effector responses is not dependent on its aminopeptidase activity, demonstrating a novel function of this protein.

### Treatment with PepN Inhibits the Initiation of TCR Signaling

**Figure 8:**
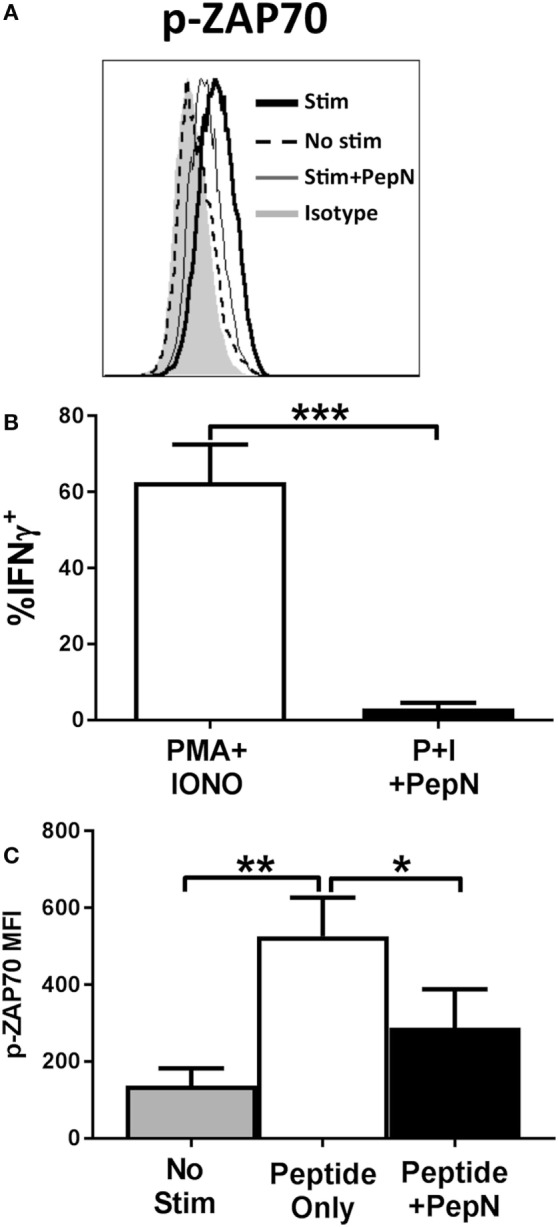
NP-specific cytotoxic T lymphocyte (CTL) treated with purified aminopeptidase N (PepN) show diminished ZAP-70 phosphorylation following peptide stimulation which cannot be restored by stimulation with phorbol 12-myristate 13-acetate (PMA) and ionomycin. NP-specific CTL were stimulated with splenocytes pulsed with 10^−6^ M NP_147–155_ peptide in the presence or absence of PepN. Following stimulation, cells were fixed with 2% paraformaldehyde to halt TCR signaling. Permeabilized cells were stained with anti-p-ZAP-70 antibody. Representative histograms **(A)** and averaged data from four independent experiments **(B)** are shown. **(C)** NP-specific CTL was stimulated with PMA and ionomycin in the presence or absence of PepN. IFNγ production under each condition is shown. Data shown are the mean ± SEM from four to seven independent experiments utilizing at least two independently generated CTL lines. *p < 0.05, **p < 0.01, and ***p < 0.001.

Given the failure to produce IFNγ, we determined whether PepN treatment impaired signaling through the TCR. One of the earliest events to occur following TCR engagement is phosphorylation of the kinase ZAP-70 [for review see Ref. (44)]. To assess activation of ZAP-70, CTL were stimulated with peptide-pulsed splenocytes for 15 min in the presence or absence of PepN at which point cells were fixed. Peptide stimulation induced a robust increase in the MFI of phospho-ZAP-70 which was inhibited in the presence of PepN (Figures 8A,B), indicating PepN treatment prevents initiation of TCR signaling.

Given the early nature of this signaling defect, we hypothesized that we could restore function through direct stimulation of downstream mediators of T cell activation using PMA and IONO. PMA and IONO directly activate protein kinase C-θ and induce Ca^+^ flux, respectively. Figure 8C shows that PMA + IONO stimulation induced a robust IFNγ response in effector cells that was blocked by the presence of PepN. The inability of PMA + IONO treatment to promote IFNγ production in the presence of PepN suggested this immunoregulatory protein also inhibited distal molecules in the TCR signaling pathway.

### Phospho-ERK1/2, Phospho-JNK, and Phospho-p38 Are Significantly Reduced following PepN Treatment

**Figure 9:**
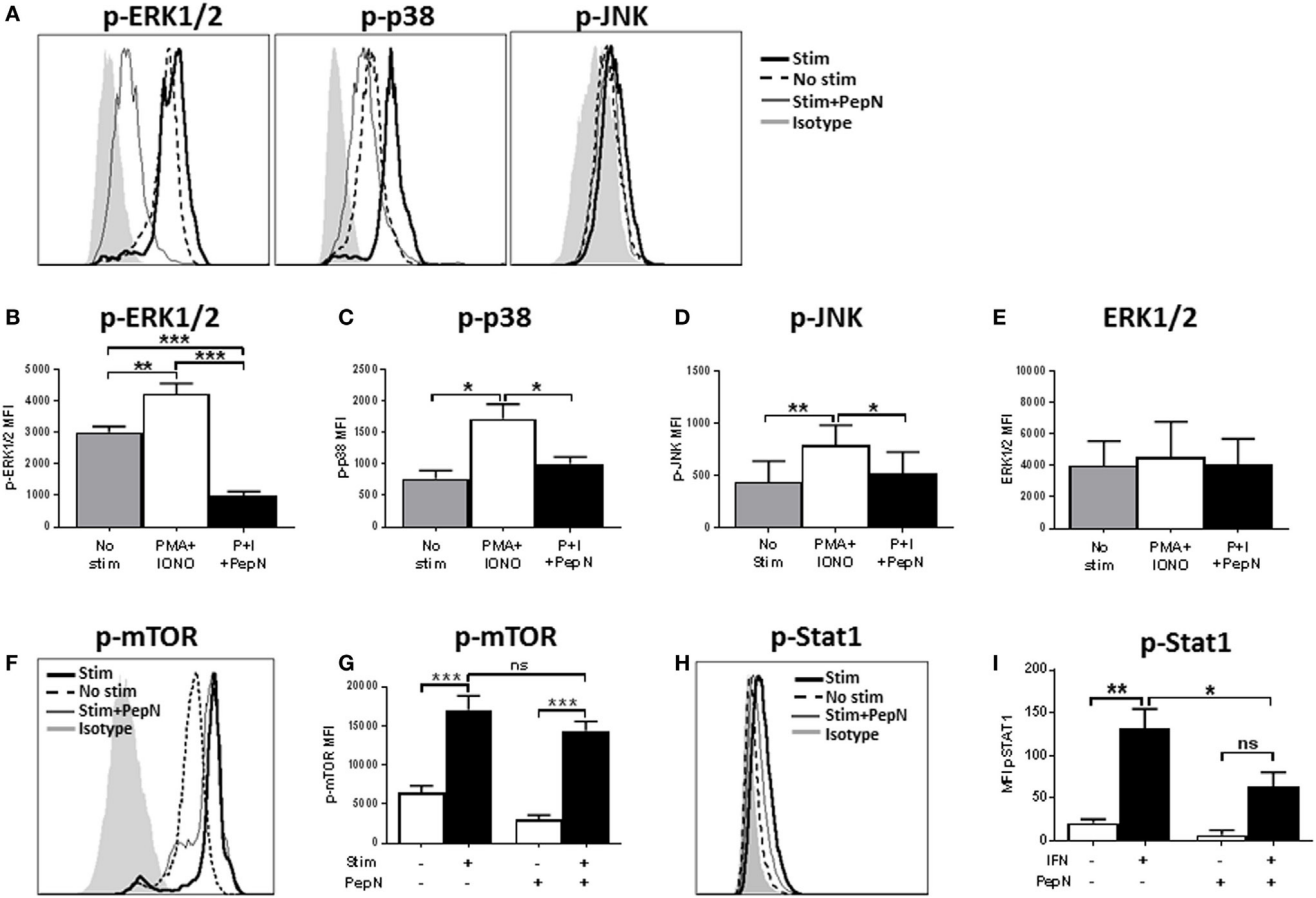
Aminopeptidase N (PepN) treatment significantly impairs many but not all molecules in the TCR signaling pathway. For analysis of TCR signaling, NP-specific cytotoxic T lymphocyte (CTL) were stimulated for 15 min with phorbol 12-myristate 13-acetate (PMA) + ionomycin (IONO) in the presence or absence of PepN followed by staining with anti-CD8 and antibody for the indicated signaling molecule. Representative histograms showing levels of phosphorylated ERK, p38, and Jun N-terminal kinase (JNK) are presented in **(A)**. The mean MFI ± SEM are shown for the level of phospho-ERK **(B)**, phospho-p38 **(C)**, phospho-JNK **(D)**, and total ERK protein **(E)**. Representative histograms **(F)** and mean MFI ± SEM **(G)** showing levels of phosphorylated mammalian target of rapamycin (mTOR). For the analysis of STAT1 activation, NP-specific CTL were stimulated for 15 min with type 1 IFN followed by staining with anti-phospho STAT1 antibody. Representative histograms are shown in **(H)** and mean MFI ± SEM in **(I)**. All data are from three to four independent experiments. *p < 0.05, **p < 0.01, and ***p < 0.001.

Given the failure of PMA + IONO to overcome the negative effects of PepN, we evaluated the signaling mediators distal to the targets of PMA and IONO action, i.e., ERK1/2, JNK, and the p38 mitogen-activated protein kinase (44). Phosphorylation of these molecules mediates the activation and nuclear translocation of transcription factors where they promote production of mRNA. We hypothesized that PepN treatment impaired activation of these molecules. To test this, CTL were stimulated with PMA and IONO in the presence or absence of PepN, followed by staining with antiphospho-ERK1/2, antiphospho-JNK, and antiphospho-p38 antibodies. We observed increases in the level of p-ERK1/2, p-p38, and p-JNK following 15 min of stimulation with PMA and IONO (representative data shown in Figure 9A and averaged data in Figures 9B–D). In contrast, CTL treated with PepN showed significantly reduced levels of these phosphorylated molecules. Interestingly, the amount of phospho-ERK1/2 in PepN-treated cells was significantly below that in non-stimulated cells (approximately threefold) (Figure 9E), suggesting the ability of PepN to modulate basal levels of p-ERK1/2. To determine whether the reduced level of phosphorylated protein was the result of protein degradation, we evaluated total ERK1/2 as a representative signaling molecule. No decrease was observed following PepN treatment (Figure 9E). Together these data show that PepN regulates T cell effector function by inhibiting phosphorylation of the TCR signaling cascade at multiple steps. This raised the interesting possibility that signaling was generally inhibited by PepN treatment.

### mTOR Phosphorylation Is Unaffected by PepN Treatment

The Akt pathway is activated downstream of signaling through TCR and is responsible for regulation of cellular metabolism. This pathway operates *via* the global regulator mTOR. When CD8^+^ T cells are activated, they are placed under metabolically intensive conditions. Glycolysis, amino acid uptake, and protein synthesis must all increase in order to facilitate appropriate effector function (45). Thus, regulation of this pathway has a profound impact on the cell. To assess mTOR activation, NP-specific CTL were stimulated for 15 min with PMA and IONO in the presence or absence of PepN as above. Following stimulation, a rapid increase in the levels of phospho-mTOR was observed (Figures 9F,G). In stark contrast to the previous signaling molecules analyzed, there was no effect of PepN treatment on mTOR phosphorylation (Figures 9F,G). These data argue that the effect on TCR signaling by PepN is not global in nature, but instead impacts selected molecules/arms of the pathway.

### PepN Treatment Can Inhibit Non-TCR Signaling Pathways

We were interested in determining whether the negative effects on signaling extended beyond the TCR signal transduction pathway. To test this hypothesis, we examined signaling through the type I IFN receptor by assessing STAT1 phosphorylation [for review see Ref. (46)]. Treatment with type 1 IFN resulted in an increase in the level of phospho-STAT1 that was significantly inhibited by PepN (Figures 9H,I). Thus, regulation of effector cells by PepN is not limited to the TCR signaling pathway.

## Discussion

In this study, we demonstrate the novel and unexpected ability of *Spn* PepN to inhibit antigen-induced effector function (cytokine production and cytolytic granule release) in T cells. To our knowledge, the ability of a bacterial aminopeptidase to inhibit T cell function has not been previously reported. Importantly, this regulatory capability was independent of its known peptidase activity. The finding that peptidase activity was not required, coupled with the inability of PMA and IONO to restore function, obviates the possibility that degradation of peptide plays a role in the inhibitory effects of PepN. Analysis of effector T cells treated with PepN revealed that the failure to produce cytokine correlated with a robust decrease in the activation of numerous molecules involved in TCR signal transduction, i.e., ZAP-70, ERK1/2, JNK, and p38.

Many bacterial species directly suppress immune signaling pathways in order to evade detection by host immune responses (40, 47). A common target of suppression is the MAP kinase pathway. This pathway is relatively conserved in nature, where organisms ranging from yeast to man use phosphorylation by protein kinases to transmit signals inter- and intracellularly. It has been estimated that ~30% of all cellular proteins can be modified by protein kinase activity (48). High reliance on this evolutionarily well-conserved signaling pathway provides ample targets for bacterial pathogens to modulate and suppress immune signaling within the host environment.

One strategy employed by pathogens to inhibit host cell signaling is to directly block activation through binding to MAP kinases (47, 49). A well characterized example of a bacterial effector protein that specifically targets TCR signaling by blocking a kinase is the *Yersinia pestis Yersinia* outer protein (Yop) J protein. YopJ is a member of the Yop family and is an acetyltransferase that directly binds to MAP-kinase-kinases, blocking both phosphorylation and subsequent activation (47). The result of this block is reduced levels of activated ERK, p38, and JNK and their associated signaling pathways (50). *Salmonella typhimurium* AvrA and *Bacillus anthracis* lethal factor employ similar strategies (51, 52).

Another mechanism for bacterial toxins to inhibit host cell signaling is by dephosphorylation of host cell signaling molecules. The *Y*. *pestis* YopH protein is a potent phosphatase that was shown to inhibit T cell activation through binding and inhibition of the adaptor molecules linker for activation (LAT) and the SH2-domain-containing leukocyte protein of 76 kDa (SLP-76), resulting in inhibition of T cell signaling (53). Similarly, *Salmonella* SpvC and *Shigella flexneri* OspF proteins are phosphothreonine lyases that target phosphorylated threonine residues on ERK and p38 leading to their inactivation (54, 55). While unknown, phosphatase activity would be an attractive hypothesis for the described inhibition of TCR signaling with PepN treatment.

Together, these examples lend credence to the model that pathogenic bacteria can target evolutionarily conserved signaling pathways, especially in immune cells. With that said, *Spn* PepN is the first example of a bacterial aminopeptidase with this activity. Interestingly, *Spn* has no described mechanism for actively delivering proteins to the host cell cytoplasm. Instead, *Spn* is known to undergo autolysis at stationary phase as a result of the action of lytA (56), which allows access of *Spn* proteins to the extracellular environment. Passive import of a protein that can inhibit host cell signaling has been reported for *Helicobacter pylori* vacuolating toxin (VacA), which can be internalized directly into the cytoplasm of host T cells through interaction with lipid rafts and cell surface receptors (57). The mechanism used by VacA to gain entry into the host T cell presents an appealing model. Such access would allow for regulation of molecules at proximal and distal steps of the pathway. An additional possibility is that PepN exerts its inhibitory effect through binding to an extracellular receptor, although it is more difficult to imagine how such an interaction regulates multiple signaling components, i.e., ERK, JNK, and p38.

It is intriguing that activated T cells express a membrane bound aminopeptidase (CD13) (58, 59). Treatment of T lymphocytes with inhibitors of CD13 activity results in inhibition of proliferation and cytokine production (60). Whether the *Spn* PepN can act as a competitor for a CD13-binding partner and thereby reduce CD13 function merits further exploration.

The newly described function of *Spn* PepN reported here has important implications for carriage and IPD. Previous work from our laboratory showed that in the context of influenza virus and *Spn* coinfection, effector cells demonstrated a reduced ability to produce IFNγ and TNFα (28). During coinfection of the lung, it is highly likely that antiviral T cells would be found in the same tissue space as invading *Spn*, allowing for potential regulation by PepN. Regulation of effector functions by *Spn* PepN could also have important consequences for bacterial clearance. CD4^+^ T cells are crucial mediators of the anti-*Spn* response (61, 62). Preliminary data suggest CD4^+^ T cells also exhibit susceptibility to inhibition by PepN. Finally, it is possible that *Spn* may not only curtail ongoing immune responses, but also negatively impact tissue resident memory T cells in the lung and nasopharynx. At present, we do not know whether PepN exposed cells can recover function. If not, modulation of tissue resident memory cell function as a result of nasopharyngeal colonization or invasion of the respiratory tract by *Spn*, could leave the host with increased susceptibility to infection following pathogen reencounter as these immune cells are critical for protection (23).

Alternatively, during carriage it is possible that low level release of PepN into the host environment could modulate the inflammatory nature of the immune environment. MAP kinase signaling is not unique to T cells; many host cells utilize this type of signaling network to produce a variety of cytokines (40, 47). This opens the possibility that *Spn* PepN modulates the inflammatory environment in the nasopharynx where the bacteria typically colonize in the absence of disease, leading to modulation of the innate response to the bacteria. Thus, the action of PepN could be broad with regard to immune regulation. Additional studies are necessary to understand the full potential of this protein in the context of *Spn* infection.

In summary, we report a novel activity for the *Spn* bacterial aminopeptidase PepN, i.e., inhibition of cytokine production and cytolytic granule release by effector T cells. Unexpectedly, inhibition was independent of the described peptidase activity of PepN. Further, the failure of T cells to produce cytokine and release cytolytic granules in the presence of PepN was associated with a robust reduction in the activation of multiple components of the TCR signaling cascade, including ZAP-70, ERK, JNK, and p38. The findings presented here elucidate a novel mechanism by which *Spn* can directly modulate host T cell effector function and may play an important role in pneumococcal disease.

## Ethics Statement

All research performed in this study complied with federal and institutional guidelines set forth by the Wake Forest School of Medicine (WFSM) Animal Care and Use Committee. The WFSM animal care and use protocol adheres to the US Animal Welfare Act and Regulations. All studies were approved by the WFSM Animal Care and Use Committee (A14-057).

## Author Contributions

LB designed the study, performed the experiments, analyzed the data, and wrote the manuscript. DP provided guidance on the study and edited the manuscript. MO performed experiments and edited the manuscript. ED performed experiments. WS provided guidance on experiments and edited the manuscript. MA supervised the project, analyzed the data, and edited the manuscript.

## Conflict of Interest Statement

The authors declare that the research was conducted in the absence of any commercial or financial relationships that could be construed as a potential conflict of interest.
